# Migraine in the multiple sclerosis prodrome: a prospective nationwide cohort study in pregnant women

**DOI:** 10.1186/s10194-024-01941-w

**Published:** 2024-12-23

**Authors:** Karine Eid, Øivind Torkildsen, Jan Aarseth, Marianna Cortese, Trygve Holmøy, Kjell-Morten Myhr, Trond Riise, Stig Wergeland, Nils Erik Gilhus, Marte-Helene Bjørk

**Affiliations:** 1 Neuro-SysMed, Department of Neurology, Haukeland University Hospital Jonas Lies Vei 71, 5053 Bergen, Norway; 2 Norwegian Centre for Headache Research, Norwegian University of Science and Technology, Trondheim, Norway; 3 Department of Clinical Medicine, University of Bergen, Bergen, Norway; 4 Department of Neurology, Haukeland University Hospital, Bergen, Norway; 5 The Norwegian Multiple Sclerosis Registry and Biobank, Haukeland University Hospital, Bergen, Norway; 6 Harvard T.H Chan School of Public Health, Harvard University, Boston, USA; 7 Department of Neurology, Akershus University Hospital, Lørenskog, Norway; 8 Institute of Clinical Medicine, University of Oslo, Oslo, Norway; 9 Department of Global Public Health and Primary Care, University of Bergen, Bergen, Norway

**Keywords:** MS prodrome, Prodromal phase, Prodromal period, Prodromal symptoms, Headache

## Abstract

**Background:**

People with multiple sclerosis (MS) have an increased risk of migraine. However, little is known about migraine and other headaches during the prodromal phase (before MS symptom onset). Our objective was to study the risk of migraine in women with MS before MS onset.

**Methods:**

A nationwide, prospective cohort study of women participating in the Norwegian Mother, Father, and Child cohort study 1999–2008. The women reported the occurrence of migraine and other headaches prior to or during pregnancy. We identified women who later developed MS through data linkage with national health registries in 2018. We excluded women with an established MS diagnosis (*n* = 125) and women who had experienced their first clinical symptom of MS, but not yet received an MS diagnosis (*n* = 91). The reference group comprised all other women in the cohort (*n* = 85,292). We used logistic regression to estimate adjusted odds ratios (aORs) with 95% confidence intervals (95% CIs).

**Results:**

Two hundred and forty-six women developed MS during follow-up. Of these, 116 women had MS symptom onset after 1–5 years, 92 after 6–10 years, and 38 after 10 years. Migraine was more common among women who developed MS compared to the reference group, 18% vs 11%, aOR 1.6 (1.2–2.3), adjusted for age, smoking, socioeconomic status and overweight. The risk of other headaches was similar for women who developed MS compared to the reference group, 29% vs 27%, aOR 1.1 (0.8–1.4). Migraine was reported by 21 of 116 (18%) women with $\le$ 5 years until MS symptom onset (aOR 1.7 [1.1–2.8]) and 19 of 92 (21%) women with 6–10 years until MS symptom onset (aOR 1.9 [1.1–2.8]. Only three of 38 (8%) women with > 10 years until MS symptom onset reported migraine, aOR 0.7 (0.2–2.2).

**Conclusions:**

Women with MS have increased risk of migraine, but not other headaches, up to a decade before the onset of classical MS symptoms. This supports that migraine can be a symptom of the MS prodrome. Special attention in people with migraine may lead to earlier recognition of MS.

**Supplementary Information:**

The online version contains supplementary material available at 10.1186/s10194-024-01941-w.

## Background

Migraine is a heterogenous disease characterized by intense headaches often accompanied by nausea, and sensitivity to light and sound [1]. Migraine can vary greatly in frequency, severity, and aura symptoms. The complex and unpredictable nature of the disease makes migraine a leading cause of disability among women.

People diagnosed with multiple sclerosis (MS) have increased occurrence of migraine compared to the general population [2–4]. Less is known about migraine before the onset of MS, such as during the MS prodrome. Risk factors for both MS and migraine include female sex, European ancestry, smoking, overweight, vitamin D deficiency, and adverse childhood experiences [5–10], and there is some genetic overlap [11]. Pathobiological mechanisms of MS may contribute to symptoms of migraine as well [12]. A study from the prospective Nurses’ Health Study found 39% increased relative risk of developing MS among those with migraine, proposing that migraine could be a risk factor for MS [13]. However, a large Mendelian randomization study found it more likely that migraine in the context of MS could be a consequence of MS, rather than a risk factor [11].

During the last decade, evidence of a prodromal period in MS has emerged from several large population-based studies [14–16]. The prodromal period is an early symptomatic stage of MS that occurs before classical MS symptoms such as optical neuritis, sensory and motor deficits. The MS prodrome consists of non-specific symptoms such as depression, insomnia, pain and fatigue up to 5–10 years before specific MS symptoms [17–19]. Migraine has been reported as a possible prodromal symptom, but without adjusting for potential confounders [16]. The few other studies that have reported that symptoms of migraine can precede MS onset or diagnosis have not been prospective [20–23], which have limited the assessment of temporality.

The occurrence of other primary headaches in MS, such as tension-type headache, seems to be similar to the general population [3, 20]. The occurrence of other headaches in the preclinical setting of MS is unknown.

Our aim was to investigate the occurrence of migraine and other headaches in women who later were diagnosed with MS, but had not yet experienced any classical MS symptoms and therefore might be in a prodromal phase of MS [19]. In this prospective and population-based study with long follow-up, we assessed the temporal relationship between migraine, as well as other headaches, and MS symptom onset, utilizing a nationwide cohort in combination with medical records and health registries.

## Methods

### Study design and population

We conducted a national, prospective cohort study using the Norwegian Mother, Father, and Child cohort (MoBa) [24]. The MoBa study included Norwegian-speaking pregnant women from all over Norway during 1999–2008. Women were invited to the MoBa study during routine ultrasound examination in pregnancy week 17–20. A total of 41% of the invited women consented to participation. During inclusion, they completed self-administered questionnaires comprising medical history, which included migraine and headache history, and demographic and socioeconomic factors.

We linked the MoBa cohort with the Norwegian Multiple Sclerosis Registry and Biobank (The MS Registry) [25] and the Norwegian Patient Registry (NPR) [26] on December 31st, 2018. This data-linkage ensured identification of all women in MoBa who developed MS after study inclusion and until the end of 2018. The MS Registry had 69% national coverage of MS cases at the time of data-linkage [27]. To identify the remaining MS cases, we linked the data to NPR. After every consultation in specialist care, NPR registration of relevant diagnoses is mandatory. The MS diagnosis in NPR have a sensitivity of 97% and a positive predictive value of 0.92 [28]. If the woman was registered in NPR with an MS diagnosis, but not in The MS Registry, we used hospital records to validate the MS diagnosis using the 2017 diagnostic criteria [29]. The MS Registry comprises information on type of MS at disease onset, date of MS symptom onset, and date of MS diagnosis. For MS cases identified through NPR, we acquired this information from the hospital records.

**Fig. 1:**
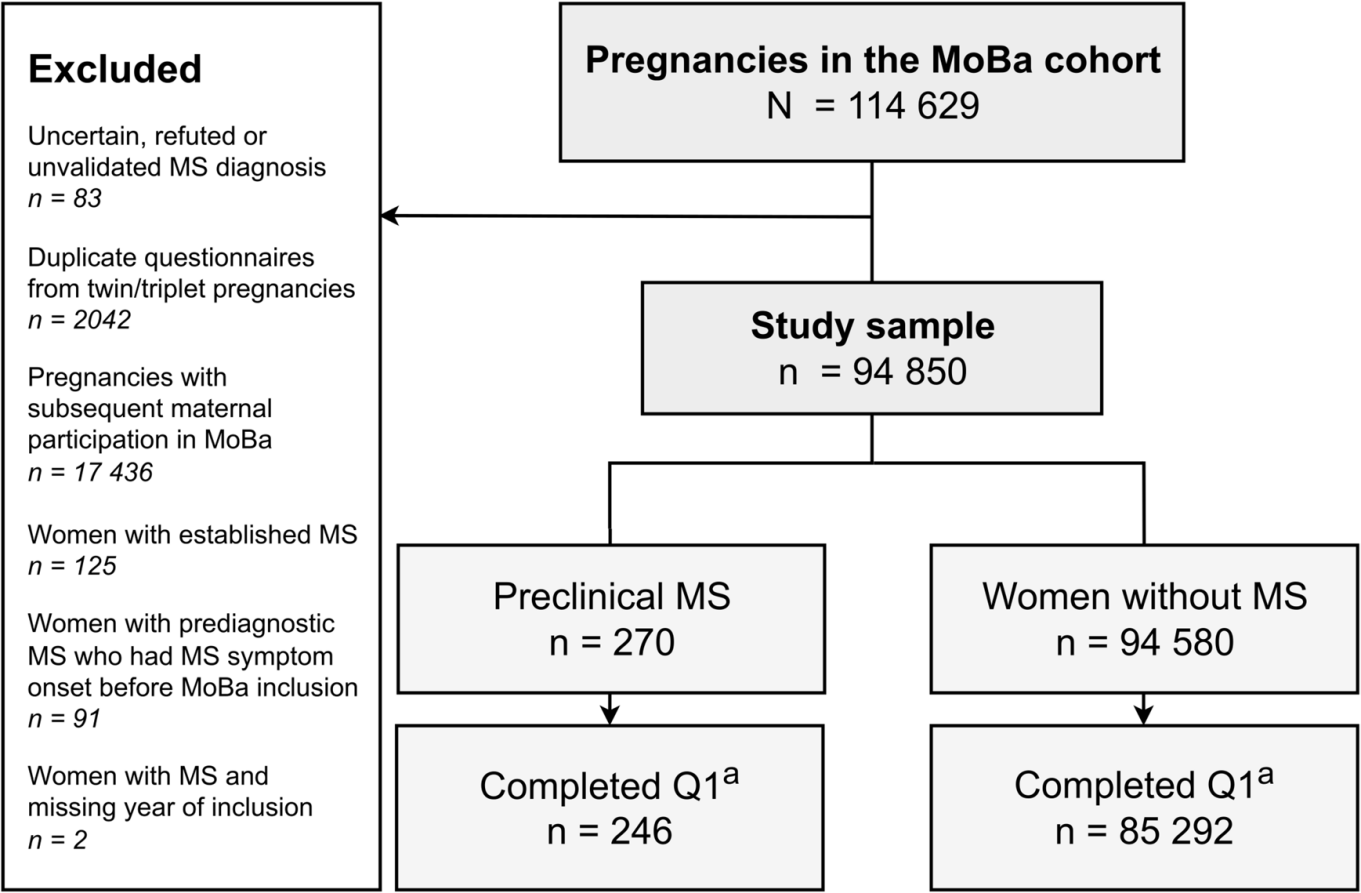
Flowchart of included and excluded participants from the MoBa cohort. MoBa = The Mother, Father and Child Cohort study. MS = Multiple sclerosis. Q1 = Questionnaire 1 in the MoBa study, received at inclusion in pregnancy week 17–20. Preclinical MS = women who developed MS after MoBa inclusion who had not yet experienced classical MS symptom onset

This study is based on version 12 of the MoBa data files, covering 114,629 pregnancies. MoBa version 12 is linked to the Medical Birth Registry of Norway, a mandatory registry comprising information on all births in Norway [30]. We excluded duplicate questionnaires due to twin or triplet pregnancies (*n* = 2042) and pregnancies where the woman had subsequent participation(s) in MoBa (*n* = 17,436) to include only one observation per woman (Fig. 1). We also excluded women with refuted, uncertain or unvalidated MS diagnosis (*n* = 83), women with established MS diagnosis prior to MoBa inclusion (*n* = 125), and women with MS with missing information on the year of MoBa inclusion (*n* = 2).

Our main study group was women who were diagnosed with MS after MoBa inclusion. To differentiate between women that could be in a prodromal stage of MS and women with early clinical but undiagnosed MS [19], we excluded women with pre-diagnostic MS who were registered with a disease onset with a classical MS symptom prior to MoBa inclusion or during the same year as MoBa inclusion (*n* = 91) (Fig. 1). The prodromal stage is considered the earliest symptomatic stage of MS and consists of unspecific signs and symptoms that occurs before the clinical stage, with more typical MS symptoms such as optical neuritis, sensory and motor deficits [19]. The duration of the prodromal phase in MS is unknown, but evidence suggests up to 5–10 years before onset of classical MS symptoms [15, 16]. Thus, we divided women with preclinical MS into three subgroups according to the time until their first classical MS symptom from study inclusion, 1) ≤ 5 years until MS symptom onset, 2) 6–10 years until MS symptom onset, and 3) > 10 years until MS symptom onset. The reference group included all women in MoBa without MS (*n* = 85,292).

### Primary outcome measure

The questionnaire in pregnancy week 17–20 (Q1) included two questions regarding whether the woman had ever experienced 1) migraine or 2) “other headache” prior to *or* during the current pregnancy. The sensitivity of self-reported migraine has not been previously validated in MoBa. Self-reported migraine has shown high agreement with the International Classification of Headache Disorders criteria in other validation studies; 87% in the population-based Women Health Study [31], and 82% in the Migraine and Pregnancy cohort study [32].

### Covariables

Relevant variables were collected through the MoBa questionnaire Q1 or through data from the Medical Birth Registry of Norway: Age at MoBa inclusion, year of childbirth, history of smoking (ever/never), overweight (pre-pregnancy body-mass index ≥ 25 kg/m^2^), non-cohabiting mother, low household income (< 60% of cohort median in the year of inclusion), short education (≤ 9 years of elementary school). Pre-pregnancy history of major depression was measured by the Lifetime major depression score [33]. Depression and/or anxiety at inclusion were measured with a combined score of anxiety and depression, a validated 5-item short version of the Hopkins symptom checklist-25 [34].

### Statistical analysis

Stata version 18 was used to make variables and to conduct the analyses (StataCorp LLC). The MS groups were compared to a reference group of women without MS. Matching was not performed. We analyzed the risk of migraine by logistic regression with estimated odds ratios (ORs) and 95% confidence intervals (CIs). Estimates with CIs not including 1 were considered statistically significant. All OR estimates were adjusted for the possible confounders age, smoking history, overweight prior to pregnancy, and adverse socioeconomic status (≥ 1 of the following: non-cohabiting mother, short education, low household income). Parity did not affect the estimates and was not included in the final model. We considered depression (current or prior) to be a potential collider on the association between migraine and MS, and depression was therefore not adjusted for in the main analyses [35]. However, depression could also potentially be a mediator between migraine and MS. We therefore additionally adjusted for prior depression and current depression/anxiety in a sensitivity analysis for the preclinical MS groups combined.

We also investigated the association between the occurrence of migraine with time until MS symptom onset by including time in years to MS symptom onset as a continuous explanatory variable in the logistic regression model. We checked the linearity assumptions by plotting the residuals from locally weighted scatterplot smoothing (lowess) curves. We illustrated the result from this analysis in a margins plot with predicted probabilities for reporting migraine for each year until MS symptom onset, using average values for the confounding variables.

### Secondary analysis

Increased prevalence of migraine and headache before MS onset could be explained by surveillance bias; that those who experience these symptoms are more likely to be examined by a neurologist and undergo brain MRI examinations, and thus more likely be diagnosed with MS. We evaluated if this affected our results by investigating age at MS symptom onset, age at MS diagnosis, and duration from MS symptom onset to diagnosis (diagnostic lag), in women pre-MS onset with migraine, in women pre-MS onset with other headaches compared to women pre-MS onset with no migraine/headache. We compared the groups with t tests when normally distributed and with the Mann–Whitney U test when data were skewed.

## Results

We included 94,850 women from the MoBa cohort in our study (Fig. 1). Median follow-up time for the cohort was 13 years (range 1–19, interquartile range (IQR) 11–15). During follow up, 270 women experienced their first symptom of MS and subsequently received an MS diagnosis. Of these, 246 women responded to the questionnaire comprising information on migraine and headache history. Among these 246 women, median time to MS symptom onset was 6 years (range 1–17, IQR 3–9), and median time to MS diagnosis was 8 years (range 1–17, IQR 5–10).

Among the 246 women who later developed MS, 116 women experienced MS symptom onset 1–5 years after inclusion in MoBa, 92 women after 6–10 years, and 38 women after 11–17 years (Table 1). Nearly all (97%) developed MS with relapsing onset. Women who developed MS were younger, more often overweight prior to pregnancy, and more often had a history of smoking, compared to women who did not develop MS. Women with less than 5 years to MS symptom onset had higher occurrence of current depression and anxiety compared to women without MS, but the occurrence of previous depression was similar.

**Table 1:** Background characteristics of women who developed MS after MoBa inclusion and women without MS

	**Preclinical MS *n* = 246**				
	**Tot MS**	**Years until MS symptom onset**			**Women without MS**
	***n* = 246**	** ≤ 5 years *n* = 116**	**6–10 years *n* = 92**	**11–17 years *n* = 38**	***n* = 85 292**
**Age; mean (SD)**	29 (5)	29 (4)	28 (5)	27 (6)	30 (5)
Missing; n (%)	0 (0)	0 (0)	0 (0)	0 (0)	5 (< 1)
**Parity; median (IQR)**	1 (0–3)	1 (0–3)	1 (0–3)	1 (0–3)	1 (0–3)
Missing; n (%)	0 (0)	0 (0)	0 (0)	0 (0)	0 (0)
**Adverse socioeconomic status**^**a**^**; n (%)**	26 (11)	8 (7)	13 (14)	5 (13)	9645 (11)
Missing; n (%)	0 (0)	0 (0)	0 (0)	0 (0)	33 (< 1)
**Depression/anxiety at study baseline**^**b**^**; n (%)**	39 (16)	23 (21)	11 (12)	5 (13)	9428 (11)
Missing; n (%)	7 (3)	6 (5)	1 (1)	0 (0)	2330 (3)
**Prior depression**^**c**^**; n (%)**	58 (24)	33 (28)	17 (19)	8 (21)	20 974 (25)
Missing; n (%)	0 (0)	0 (0)	0 (0)	0 (0)	0 (0)
**Ever smoker; n (%)**	151 (62)	67 (58)	56 (62)	28 (74)	43 830 (52)
Missing; n (%)	2 (< 1)	1 (< 1)	1 (1)	0	560 (< 1)
**BMI ≥ 25 kg/m**^**2**^**; n (%)**	93 (39)	43 (39)	37 (41)	13 (36)	26 064 (31)
Missing; n (%)	9 (4)	6 (5)	1 (1)	2 (5)	2265 (3)
**Age at MS symptom onset**^**d**^**;** **mean (SD)**	35 (6)	33 (5)	36 (5)	40 (6)	N/A
Missing; n (%)	0 (0)	0 (0)	0 (0)	0 (0)	N/A
**Age at MS diagnosis; mean (SD)**	36 (5)	35 (5)	37 (5)	41 (6)	N/A
Missing; n (%)	1 (< 1)	0 (0)	0 (0)	1 (3)	N/A
**Type of MS at disease onset; n (%)**					N/A
Relapsing-onset Progressive-onset Uncertain	238 (97) 2 (< 1) 6 (2)	114 (98) 0 (0) 2 (2)	88 (96) 1 (1) 3 (3)	36 (95) 1 (3) 1 (3)	

*Abbreviations*: *MS* Multiple Sclerosis, *MoBa* The Norwegian Mother, Father, and Child cohort study, *SD* Standard Deviation, *BMI* Body mass index, *N/A* Not Applicable. Preclinical MS = women who developed MS after MoBa inclusion who had not yet experienced classical MS symptom onset

^a^ Adverse socioeconomic status is one of the following: non-cohabiting mother, short education ≤ 9 years or low household income (< 60% of the study population median in the given enrollment year)

^b^ Depression and/or anxiety was measured by a combined score of depression and anxiety (Hopkins Symptom Checklist-5) from the inclusion questionnaire in MoBa

^c^ Lifetime depression screened by Lifetime History of Major Depression Score

^d^ MS symptom onset is the first clinical symptom of MS

A total of 18% of the 246 women who developed MS had ever experienced migraine compared to 11% of the women without MS (Table 2). The crude OR was 1.7 (95% CI 1.2–2.3), and 1.6 (95% CI 1.2–2.3) after adjusting for age, smoking, socioeconomic status and overweight. The association persisted with additional adjustment for previous depression and current depression and/or anxiety: aOR 1.5 (95% CI 1.1–2.2). The risk of other headaches was similar for women who developed MS compared to the women who did not develop MS, 29% vs 27%, aOR 1.1 (95% CI 0.8–1.4).

**Table 2:** Migraine and headaches among women who developed MS after MoBa inclusion and women without MS

	**Migraine**			**Other headaches**		
	**n (%)**	**aOR (95% CI)**	***P* value**	**n (%)**	**aOR (95% CI)**	***P* value**
**Preclinical MS** ***n*= 246**						
*Years until MS symptom onset*	43 (18)	1.6 (1.2–2.3)	0.006	71 (29)	1.1 (0.8–1.4)	0.63
≤ 5 years (*n* = 116)	21 (18)	1.7 (1.1–2.8)	0.029	35 (30)	1.2 (0.8–1.7)	0.50
6–10 years (*n* = 92)	19 (21)	1.9 (1.1–3.2)	0.016	26 (28)	1.1 (0.7–1.7)	0.79
> 10 years (*n* = 38)	3 (8)	0.7 (0.2–2.2)	0.53	10 (26)	0.9 (0.4–1.9)	0.72
**Women without MS** *n* = 85 292	9718 (11)	1.0 (ref.)	Ref	22 939 (27)	1.0 (ref.)	Ref

*Abbreviations*: *MS* Multiple sclerosis, *MoBa* The Norwegian Mother, Father and Child Cohort study, *aOR* adjusted Odds Ratios, *CI* Confidence Interval. Preclinical MS = women who developed MS after MoBa inclusion who had not yet experienced classical MS symptom onset. Women with preclinical MS were compared to women who did not develop MS during follow-up. ORs are adjusted for age, smoking history, socioeconomic status, and overweight

### Time until MS symptom onset

Migraine was reported by 18% of women with $\le$ 5 years to MS symptom onset, and by 21% of women with 6–10 years until MS symptom onset (Table 2). The aORs were 1.7 (95% CI 1.1–2.8) and 1.9 (95% CI 1.1–3.2), respectively. Women with > 10 years until MS symptom onset did not have increased risk of migraine, aOR 0.7 (95% CI 0.2–2.2).

**Fig. 2:**
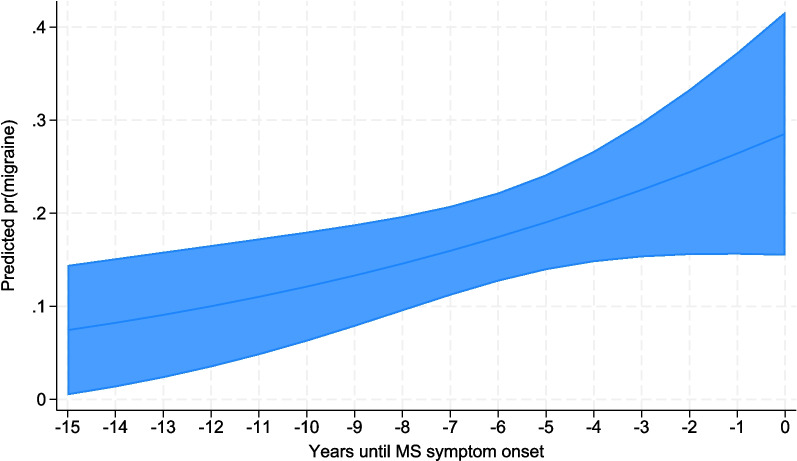
Predicted probabilities of migraine by year until MS symptom onset. Margins plot visualizing average predicted probabilities of migraine calculated from the logistic regression model using time until MS symptom onset as a continuous variable. Adjusted for age, smoking, socioeconomic status, and overweight

Using time until MS symptom onset as a continuous variable, the odds of reporting migraine increased with 12% per year closer to MS symptom onset, aOR 1.12 (95% CI 1.0–1.3). The increasing risk of migraine per year to MS symptom onset is illustrated in Fig. 2.

### Secondary analysis

The age at MS symptom onset and MS diagnosis was similar for women who developed MS that reported migraine compared to women who developed MS who did not report migraine or headache (Table 3). The time from MS symptom onset to MS diagnosis (diagnostic lag) was 1.7 years in women with migraine, and 1.5 years in both women with other headaches, and in women with no report of migraine or other headaches.

**Table 3:** Age of MS onset, diagnosis, and diagnostic lag in women with MS by headache status

	**Women with preclinical MS**							
	**Migraine + *n* = 43**			**Other headaches + *n* = 71**			**No migraine or headache (ref.) *n* = 146**	
	**Mean (SD)**	**Range**	**P value**	**Mean (SD)**	**Range**	**P value**	**Mean (SD)**	**Range**
**Age at MS symptom onset (years)**	33.6 (5.4)	24–48	0.12	34.8 (5.7)	23–50	0.72	35.1 (5.4)	20–49
**Age at MS diagnosis (years)**	35.3 (5.3)	25–49	0.16	36.3 (5.8)	25–52	0.73	36.6 (5.2)	26–49
**Time from MS symptom onset to diagnosis (years)**	1.7 (2.0)	0–9	0.06	1.5 (2.1)	0–11	0.47	1.5 (2.1)	0–8

Women with preclinical MS and I) migraine or II) other headaches were compared to a reference group of women with preclinical MS with no report of either migraine or headache. *P* values were calculated from t tests when normally distributed and skewed data with the Mann–Whitney U test. Range values are presented as minimum to maximum values

*Abbreviations*: *MS* Multiple sclerosis, *SD* Standard Deviation, *Preclinical MS* women who developed MS after MoBa inclusion who had not yet experienced classical MS symptom onset

## Discussion

In this nationwide, prospective cohort study, we found that women who later developed MS had higher odds of experiencing migraine up to 10 years prior to classical MS symptom onset, compared to women that did not develop MS during follow-up. The risk of migraine gradually increased closer to MS symptom onset. This supports that migraine could be a symptom of the MS prodromal period. MS prodromes consist of unspecific symptoms in the 5–10 years preceding MS symptom onset, with an increasing burden of medical complaints closer to MS onset [15, 16].

Our use of a prospective and population-based design extends previous knowledge on the temporal relationship between migraine and MS. A few studies have reported that migraine precedes MS onset or diagnosis by 7–8 years on average [21, 22]. However, these studies have been limited by small case numbers, retrospective study designs and lack of precise information on MS symptom onset. A study from the prospective Nurses’ Health Study found that 19% of nurses who later developed MS reported migraine at baseline [13], which is similar to 18% in our cohort.

We were able to adjust for important confounders, including smoking, overweight and socioeconomic status. These environmental factors are associated with both increased risk of migraine and of developing MS [5, 7, 8]. This suggests that migraine in the prodromal setting of MS may be associated with MS-specific factors. Neuroinflammation is a key part of MS pathophysiology and does also play an important role in migraine [36]. The pathobiological mechanisms of MS probably start many years before the first evident symptom. Neurofilament light, a biomarker for neuroaxonal damage, increases in blood up to a decade before MS symptom onset [37, 38].

Current depression and anxiety were more frequent among women with less than 5 years until MS symptom onset compared to women who did not develop MS, whereas the history of previous depression was similar. Psychiatric morbidity has been suggested as a MS prodromal feature by several studies [15–18]. Depression, anxiety and stress are associated with several pain syndromes, including migraine and other headache etiologies [12]. We were able to adjust for both previous depression and current depression or anxiety in a sensitivity analysis. The higher frequency of migraine in the MS prodrome was not explained by depression or anxiety.

The risk of reporting non-migraine headaches was not increased in women before MS onset compared to women who did not develop MS. Non-migraine headache was more frequent than migraine in both groups. Tension-type headache is the most common headache in the general population [39]. Most previous studies have not found any increased risk of tension-type headache among people with established MS [3]. The occurrence of tension-type headache in the preclinical setting of MS has not been examined previously.

Headache is the most common reason for being referred to an MRI in radiologically isolated syndrome [40], potentially leading to an earlier diagnosis of MS in people with headache compared to those without headache. However, surveillance bias did not have a major impact on our results. First, we found no difference in age at MS symptom onset, age at diagnosis, or in time from symptom onset to MS diagnosis between women with and without migraine. Second, the risk of migraine was increased as long as 10 years before the first symptom of MS. Third, we did not find any association between preclinical MS and other headaches, which, similarly to migraine, are frequent indications for MRI [41].

Strengths of our study include a large, well-characterized, and prospective population-based cohort with data-linkage that ensured validated MS diagnoses and long follow-up. We had the possibility to identify women before the clinical stage of MS due to precise information on date of MS symptom onset. This enabled us to study the temporal relationship between migraine and MS. Moreover, we were able to adjust for important factors that may have confounded or mediated the relationship between MS and migraine.

There are some limitations to our study. Migraine and other headaches were self-reported which could result in misclassification. However, this potential misclassification would be non-differential between people with preclinical MS and the control group and would not lead to bias. We lacked nuanced information on headache symptoms, such as frequency of attacks, date of onset, if migraine was with/without aura, or if “other headaches” implied tension-type headache or other headache types. We were unable to do nuanced analyses of how pregnancy-related factors may have modified the risk of migraine. Further research that includes detailed information on migraine symptoms and their onset is needed to investigate whether there exists specific characteristics of migraine in the MS prodrome, and for a more in-depth understanding of the temporal relationship between migraine and MS. The sample size was small for women with more than 10 years to MS symptom onset. The participants were only female, pregnant, and Norwegian speaking, which may limit generalizability to other populations.

## Conclusions

The increased occurrence of migraine among women in the prodromal phase of MS shown in this study, implies that clinicians should give special attention to people with migraine with symptoms or heightened risk of MS, to ensure timely recognition and treatment. Symptoms of MS may overlap with intermittent symptoms related to migraine. Visual disturbances, pain in the orbital area, paresthesia, dizziness and fatigue are common both in MS and among people with migraine. Furter, some studies have reported increased occurrence of white matter lesions among people with migraine [42]. It is important that clinicians do not dismiss potential MS symptoms among these patients. Early intervention in MS leads to slower disease progression and better long-term prognosis.

## Supplementary Information

Supplementary Material 1.

Supplementary Material 2.

## Data Availability

Data may be obtained from a third party and are not publicly available. Access to data from MoBa can be obtained through an enquiry to the Norwegian Institute of Public Health. Data from the MS Registry are accessible for researchers by application.

## Abbreviations

aOR: Adjusted odds ratio
CIs: Confidence intervals
IQR: Interquartile range
MS: Multiple sclerosis
MoBa: The Norwegian Mother, Father, and Child cohort study
NPR: The Norwegian Patient Registry
The MS Registry: The Norwegian Multiple Sclerosis Registry and Biobank
