# Supplementary figures and images for: Migraine in the multiple sclerosis prodrome: a prospective nationwide cohort study in pregnant women
Source: J Headache Pain. 2024 Dec 23;25(1):225. doi: 10.1186/s10194-024-01941-w (PMC11665115; doi:10.1186/s10194-024-01941-w)

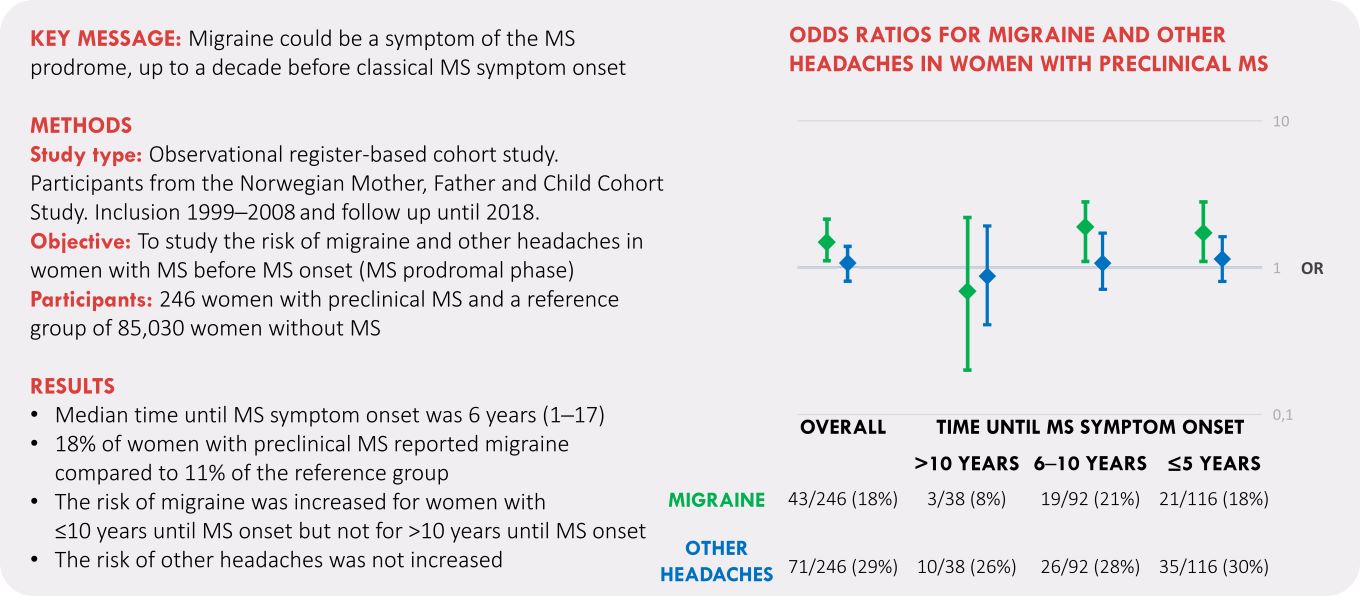

Supplement: Supplementary file 2 — Supplementary Material 2. [file 10194_2024_1941_MOESM2_ESM.jpg]
